# Supplementary material for: Machine learning and deep learning techniques to support clinical diagnosis of arboviral diseases: A systematic review
Source: PLoS Negl Trop Dis. 2022 Jan 13;16(1):e0010061. doi: 10.1371/journal.pntd.0010061 (PMC8791518; doi:10.1371/journal.pntd.0010061)
Supplement: Attachment — Submitted filename: PNTD - Reviewers Responses to Questions.pdf [file pntd.0010061.s001.pdf]

Reviewer's Responses to Questions

We would like to thank you for all your comments and suggestions made to improve the quality of our work. We have carefully read and addressed them. All modifications are highlighted in blue in the revised version of the manuscript.

Key Review Criteria Required for Acceptance?
As you describe the new analyses required for acceptance, please consider the following:

Methods
-Are the objectives of the study clearly articulated with a clear testable hypothesis stated?
-Is the study design appropriate to address the stated objectives?
-Is the population clearly described and appropriate for the hypothesis being tested?
-Is the sample size sufficient to ensure adequate power to address the hypothesis being tested?
-Were correct statistical analysis used to support conclusions?
-Are there concerns about ethical or regulatory requirements being met?

Reviewer #1: (No Response)

Reviewer #2: My main concern is why authors decide to perform the systematic review only by searching studies in one database, “Google Scholar”, and not including other databases, i.e. three significant publishers (PubMed, Springer and IEEE).
Do the authors select this database for a specific reason?. Please clarify and explain why you decide not to include more databases in the search.

Answer: A recent comparative study of 12 commonly used academic search engines and bibliographic databases suggests that Google Scholar is currently the most comprehensive academic search engine (Gusenbauer, 2019) [1]. Initially, we actually used more publisher-specific search engines including those you cite. However, as part of our own internal assurance process, we also generated a dataset from Google Scholar and found that in addition to those in PubMed, Springer, IEEE and others, Google Scholar identified additional publications. We have added clarifying text in the body of the revised transcript to this effect.

[1] Gusenbauer, M., 2019. Google Scholar to overshadow them all? Comparing the sizes of 12 academic search engines and bibliographic databases. Scientometrics, 118(1), pp.177-214.

Results
-Does the analysis presented match the analysis plan?
-Are the results clearly and completely presented?
-Are the figures (Tables, Images) of sufficient quality for clarity?

Reviewer #1: (No Response)

Reviewer #2: The authors perform exceptional work reviewing literature and explaining ML concepts to non-expert audiences interested in reading the journal (Focused on tropical infectious diseases). I suggest including the explanation of some concepts that are mentioned through the review that may help the reader to understand some points. I.e., Grid search, hyperparameters, Gamma parameters, Model overfitting.
Answer: We have explained some of these concepts in sub-section 3.3.

Conclusions
-Are the conclusions supported by the data presented?
-Are the limitations of analysis clearly described?
-Do the authors discuss how these data can be helpful to advance our understanding of the topic under study?
-Is public health relevance addressed?

Reviewer #1: (No Response)

Reviewer #2: The authors exposed the results obtained and formulated a reflection about needs in the area and how the application of ML would be beneficial to the study and diagnosis of arbovirus diseases. Moreover, they exposed the caveats in the area and the urgent needs to address in future studies.
Answer: Thank you very much.

Editorial and Data Presentation Modifications?
Use this section for editorial suggestions as well as relatively minor modifications of existing data that would enhance clarity. If the only modifications needed are minor and/or editorial, you may wish to recommend “Minor Revision” or “Accept”.

Reviewer #1: (No Response)

Reviewer #2: Due to the nature of the review and the non-expert in ML audience expected in a journal focused on infectious diseases, I suggest integrating concepts to explain the ML methods in a more organised way, first specify that the methods discussed are supervised methods, and explain what is Deep learning.
Answer: We added new introductory content about AI, ML, supervised learning and DL in sub-section 3.2 of the revised manuscript.

- 3.2.3 Section (Neural Networks) authors start explaining MLP with no introduction and may seem a bit confusing.
Answer: We added background text on MLP in sub-section 3.2.3 of the revised manuscript.

- Some abbreviations are missing in the list (AI, XAI, MOMO%), and others are doubly explained (SLR).
Answer: We have revised the entire manuscript and added the abbreviations that were missing.

Summary and General Comments
Use this section to provide overall comments, discuss strengths/weaknesses of the study, novelty, significance, general execution and scholarship. You may also include additional comments for the author, including concerns about dual publication, research ethics, or publication ethics. If requesting major revision, please articulate the new experiments that are needed.

Reviewer #1: The authors review published studies using machine learning to predict arboviruses from clinical and laboratory data. Overall, they do a nice job of collecting the existing research, are rightfully critical of some of some of the major shortcomings of studies in this field including, overfitting, the selection of evaluation metrics and the poor documentation of code, data and methods.
Answer: Thank you for the review and comments. We have read and addressed them carefully.

Tables 3 and 4 provide a very nice summary of the features used in the studies.

In section 3.3 I was pleased to see the authors flagging studies that appear to be overfitting or inappropriately benchmarking their studies as this practice is unfortunately common in biological applications of supervised machine learning. It would be great if they explicitly flagged these studies in table 5. A discussion about the importance of regularization method like batch normalization, L1, L2 and C in SVM would have also been helpful. Many readers may not understand the importance of this for generalization.
Answer: We have updated Table 5 and added a discussion about the importance of regularization methods. We have also improved the presentation of basic concepts of ML and DL to support readers who may not be as familiar with ML/DL (please see sub-section 3.3).

Throughout the paper they refer to Machine learning and (ML)and Deep learning (DL) separately. DL is a subset of ML and only one study referred to DL so it would be much clearer to just refer to everything as ML except when specifically talking about the Ho et al. (2020) paper.
Answer: We have added new text about AI, ML, supervised learning and DL in sub-section 3.2 of the revised manuscript.

The review spends a lot of time discussing the use of a convolutional neural network (CNN), DenseNet, by Ho et al. (2020). They discuss CNN for a half a page on page 9 and again on page 10 and page 24. That model did not perform appreciably better than a logistic regression or random forest model. DenseNet is unique in that it connects each layer to every other layer in a feed forward neural network and essentially concatenates features rather than summing them. But like other CNN's it

relies on pooling and convolutions at different scales. While this makes sense for image data where the proximity of pixels has meaning, it is seemingly meaningless for the 16 features clinical and laboratory features placed in a random order in the Ho et al. (2020) analysis. It seems that DenseNet sort of just devolves into a multilayer perceptron or similar since the convolutions are meaningless. It would be good to point this out. More generally, In the discussion the authors make the recommendation to invest in CNN, LSTM. The data types used in the model are not spatially or relationally structured and the amount of training data do not lend themselves well to these large models. I have concerns about this recommendation and the use of recurrent models (RNN, CNN, and LSTM) generally on this kind of data.
Answer: Thank you for the comment regarding the usage of CNN for solving tabular data problems. We have added a discussion in sub-section 3.3.1 of the revised manuscript about this topic. We also revised the discussion in Section 4.

Our findings suggest that previous works done on classification of arboviral diseases mostly use tabular data and are dominated by tree-based models. To make use of tabular data with DL models, the main challenge is to reshape the data to fit it in the specific input representation. Commonly, datasets are high-dimensional and very sparse, and then the difficulty of reshaping a good input representation is magnified [2]. Another challenge for DL models working with tabular data is related to the scale and distribution of the features present in the dataset. For tree-based models, this aspect hardly matter; on the other hand, DLs are very sensitive to them, and can cause vanishing and exploding gradient problems [2].

Despite these challenges, we can take advantage of powerful features that DL can inherently provide. For instance, by using a DL model, we can reduce the manual feature engineering, which is a time-consuming task commonly used when pre-processing the dataset for ML training and testing, by using convolution and max pooling layers. Some works have proposed the transformation of tabular data to images (matrix) in order to feed them into a DL model, such as Alvin et al. [3] that proposed a CNN and a LSTM to detect sepsis in neonates. Their results show that DLs outperformed some traditional ML models, such as SVM and logistic regression, proving that significant improvements can be done with DL if data is reshaped accordingly. Future research should consider benchmarking the performance of a wider set of DL architectures, both discretely and as part of ensembles, including RNNs, DBNs, and DSNs, amongst others, using different techniques for reshaping the dataset. In particular, the use of LSTM and GRU, both types of RNNs, may prove fruitful.

[2] Marais JA. Deep learning for tabular data: an exploratory study. Stellenbosch; Stellenbosch University; 2019.
[3] Alvi RH, Rahman MH, Khan AAS, Rahman RM. Deep learning approach on tabular data to predict early-onset neonatal sepsis. Journal of Information and Telecommunication. 2020; p. 1–21.

I appreciated that the authors highlighted the issue of imbalanced datasets in the studies reviewed. I think this could bear even more discussion. It could also be expanded to the larger issue of dataset shift problems like prior probability shift and

covariance shift. I think there is likely to be major issues with the ability of these models to generalize well.
Answer: We have added a discussion in Section 4 of the revised manuscript about issues relating to imbalanced datasets, the dataset shift problem and generalization.

It would also be worth discussing if the studies point to general limits to the approach. Given the small number of general features it may be that 85% accuracy is about the best that can be expected. Given that this is data predominantly comes from a population where a healthcare professional suspected an arboviral infection and ordered a clinical test, a classifier with this level of accuracy may not perform better than a clinician assessing a patient with symptoms. This would suggest that it would be better to investing resources in developing low-cost clinical diagnostics with the potentially to achieve much higher accuracy.
Answer: We have added a discussion in Section 4 of the revised manuscript.

The authors point to the lack of multiclass classification algorithms, this is a good point that could be expanded. Differential diagnostic procedures are employed by clinicians to diagnose patients, but it is not clear that any of these models considered a representative range of febrile illnesses.
Answer: We have expanded the discussion about multi-class problems in Section 4 of the revised manuscript.

Smaller items:
- P3 line 4: “weeks years” should probably read “weeks to years”

Answer: Done. Thank you.

- Section 3 seems like a pretty general overview of ML classifiers more than an arbovirus specific review, perhaps this could be shortened.

Answer: This section was included to support the needs of the wider audience for the journal and is consistent with other reviewer feedback. We are happy to further edit back introductory content if reviewers and editors require it.

- Reverse snowballing should be explained

Answer: We have explained reverse snowballing in sub-section 2.1 of the revised manuscript.

Reviewer #2: Authors perform a systematic review to elucidate the usage of machine learning techniques in the diagnosis, classification and study of arboviral infectious diseases. The authors made an effort to explain ML techniques to non-expert readers and explore some perspectives related mainly to the usage of these technologies. The work is well presented and treat a topic of interest for the journal.
Moreover, a non-expert in ML reader would understand the article due to the precise and current representation of the scientific topic selected.
My suggestion is to accept the manuscript, and my comments are related to clarifying some concepts to give strength to the review by helping non-expert readers understand the topic.

In the conclusion section, the authors suggest future directions and open challenges to set the basis of subsequent studies applying ML to the diagnosis of arboviruses diseases.

Answer: We do appreciate the comments. We have revised the entire manuscript and addressed all suggestions in order to improve the quality of our work. Thank you.
